# Changing Human-Animal Relationships in Sport: An Analysis of the UK and Australian Horse Racing Whips Debates

**DOI:** 10.3390/ani6050032

**Published:** 2016-05-03

**Authors:** Raewyn Graham, Phil McManus

**Affiliations:** School of Geosciences, The University of Sydney, New South Wales 2006, Australia; phil.mcmanus@sydney.edu.au

**Keywords:** thoroughbred horse racing, whip, human-horse relations, media framing, print and social media

## Abstract

**Simple Summary:**

This paper identifies the various perceptions held by advocates and opponents about the use of the whip in thoroughbred racing, as portrayed in print and social media. Three time periods in two countries were investigated, Australia (2009) and the UK (2011), following the introduction of new whip rules and the period August 2014–August 2015 for both countries to identify whether perceptions had changed. The major area of contention between advocates and opponents was whether the whip is an essential tool or a cruel instrument. This tension remained in 2015. The research also revealed that people opposed to the whip were more likely to express their views on social media than in print media.

**Abstract:**

Changing social values and new technologies have contributed to increasing media attention and debate about the acceptable use of animals in sport. This paper focuses on the use of the whip in thoroughbred horse racing. Those who defend its use argue it is a necessary tool needed for safety, correction and encouragement, and that it does not cause the horse any pain. For those who oppose its use, it is an instrument of cruelty. Media framing is employed to unpack the discourses played out in print and social media in the UK (2011) and Australia (2009) during key periods of the whip debate following the introduction of new whip rules. Media coverage for the period August 2014–August 2015 for both countries is also considered. This paper seeks to identify the perceptions of advocates and opponents of the whip as portrayed in conventional and social media in Australia and the UK, to consider if these perceptions have changed over time, and whose voices are heard in these platforms. This paper contributes to discussions on the impacts that media sites have either in reinforcing existing perspectives or creating new perspectives; and importantly how this impacts on equine welfare.

## 1. Introduction

Recent media coverage of the live-baiting scandal in Australian greyhound racing highlights how society’s view on what is an acceptable use of animals in sport has changed. This change in social values has seen an increase in public awareness of a multitude of animal welfare issues and what “constitutes a defensible use of animals” in sports ([1], p. 204). Horse racing is not immune to these changes. Arthur makes the salient point that, “there is a realization that society may not tolerate horse racing much longer unless racing becomes safer and kinder to the horse. Horse racing must change to survive” ([2], p. 242). Two key factors have influenced changes in the ways in which racehorses are valued. One factor, cultural shifts have seen the views of animal rights enter mainstream consciousness [3]. From the late 1970s onwards a number of animal rights organizations have formed to oppose the exploitation of animals. One of the largest animal protection groups in the UK, Animal Aid, was formed in 1977; the international organization People for the Ethical Treatment of Animals (PETA) was established in 1980; and in 2008 the Coalition for the Protection of Racehorses (CPR, Australia) began their campaigns to “address serious welfare concerns” in the horse racing and breeding industry [4].

The second factor is that communication technology has transformed how horse racing is mediated and how associated welfare issues are debated in the media. Televised coverage of race meetings has opened the sport to a global audience, with supporters and proponents of racing now able to view races on a variety of social media devices [1]. Racing, therefore, no longer occurs only at a racecourse, attended by local racing enthusiasts. Images are now able to be stored for indefinite periods on the internet, meaning that that “nothing happens on the track any longer without some form of documentation and information storage” ([5], p. 141). Public interest in animal welfare has been influenced by media coverage of racing events. For example, the image of American Pharoah being whipped 32 times during the running of the 2015 Kentucky Derby was transported into the private spheres of millions of people, creating intense debate in the media about the appropriate use of the whip in horse racing. Currently there are no limits to the number of times a whip can be used in thoroughbred racing in Kentucky. The rules of racing in the USA differ by racing jurisdictions, but some follow guidelines established by the Association of Racing Commissioners International (ARCI), which state that whip “use that is deemed to be brutal or excessive is prohibited” [6]. Thoroughbred racing occurs in more than 50 countries, all of which have their own whip rules. Of those, Norway is one country that has successfully implemented whip-free racing in the majority of their thoroughbred races. In 1982, the use of the whip was restricted so that it could only be carried for safety. These measures were strengthened in January 2009 when the use of the whip was forbidden in most races [7]. Although, it should be noted that Norway is a relatively minor participant in global thoroughbred racing. In 2014, there were 258 races held in Norway compared with 6308 in Great Britain and 19,427 in Australia (these figures exclude jumps races) [8].

Welfare concerns associated with horse racing are therefore now global issues, with diverse groups involved in debates about the appropriate care of thoroughbred horses. Animal rights and welfare organizations are key actors in the debate, but they adopt different approaches to horseracing. The Royal Society for the Prevention of Cruelty to Animals (RSPCA), one of the leading animal welfare organizations in the world, considers thoroughbred racing as “permissible provided they (the racing industry) conform to agreed standards of stewardship and animal welfare” ([5], p. 142). For the Coalition for Protection of Racehorses (Australia), PETA (worldwide) and Animal Aid (UK), organizations that define themselves at animal rights groups, horseracing is a cruel and exploitative sport. It is, however, only Animal Aid that specially calls for an “end to commercial racing” [9]. These three groups and the RSPCA (Australia) are of the opinion that the whip should be banned. RSPCA (UK) adopts a different position arguing that the whip can be used provided that its use is “reduced to the minimum needed to ensure horse and jockey safety” and that whip rules are enforced [10]. 

This paper focuses on the debates about the use of the whip in thoroughbred racing playing out in mass media. Those who defend its use argue that the whip is a necessary tool needed for safety, correction and encouragement, and that it does not cause the horse any pain. For those who oppose its use, the whip is an instrument of cruelty. A body of work in veterinary literature has investigated how useful the whip is as a performance aid [11], and questioned the pain-free status of the padded whip [12]. Jones *et al.* (2015), in their recent critique of the British Horseracing Authority (BHA) (2011) report, *Responsible Regulation: A Review of the Use of the Whip in Horseracing*, similarly dispute claims that the whip is pain-free [13]. To date, there has been a relative lack of critical social scientific engagement with horse whipping controversies. This paper addresses this neglect by employing framing analysis to unpack discourses played out in print and social media (online readers’ comments from responses to newspaper articles and other online content) in the UK and Australia during recent key periods of the whip debate.

The time periods considered are April 2011–February 2012 in the UK, following the release of the BHA (2011) report, the introduction of new whip rules and subsequent amendments. In Australia, the key period was 2009 March–September, following the introduction of the padded whip and new whip rules. Media coverage for the period August 2014–August 2015 for both countries is also considered to ascertain what, if anything, has changed since the new rules were introduced. The five research questions are: (1) What are the perceptions of advocates and opponents of the whip as portrayed in conventional and social media in Australia and the UK? (2) Do these perceptions change over time? (3) Whose voices are heard in these platforms? (4) What impact do these media sites have in reinforcing existing perspectives or creating new perspectives; and importantly, (5) what does this mean in relation to equine welfare?

This paper begins by describing the new whip rules in the UK and Australia, and subsequent amendments. The relevant literature about human-equine relations and media framing is then discussed. The methods utilised are then detailed, before presenting the media frames and the differences between countries, media platforms and time periods. In the following section, the perceptions regarding the use of the whip are discussed, as is the impact this has on equine welfare.

From an animal rights perspective the only solution is that the whip is banned. Calls for such a ban are also coming from people inside the racing industry. At the 2015 World Horse Conference, John Francome, an ex-jockey who “rode more than 1100 winners” and current author, has reiterated his stance that the industry needs to stop using the whip. In his words:

Does it (the whip) look good? Definitely not. And what are the positives of not having a whip? Jockeys would have to keep both hands on the reins and work a lot harder. It’s about 20 percent harder to ride a finish without using your whip … they would have to think more.

[14]

If an ex-jockey of this calibre considers it is time for change than may be the racing industry needs to have contingency plans which consider the real possibility of whip-free racing.

## 2. The Introduction of the UK (2011) and Australian (2009 and 2015) Whips Rules and Their Amendments

The introduction of new whip rules in the UK and Australia created intense debates in the media about the appropriateness of the use and regulation of the whip. In the UK new rules introduced in September took effect on 10 October 2011. The UK had introduced padded whips in 2004 for jumps racing and in 2007 for flat racing. These replaced the older style whip which many (inside and outside of the racing industry) recognized was harmful to horses. In Australia, the padded whip and the new whip rules were implemented on 1 August 2009.

The key changes that caused the most concern to UK jockeys were related to the number of times the whip could be used and the fines and suspensions imposed. The new rules restricted the total number of times the whip could be used to seven times in a flat race, with a maximum of five of those in the last furlong (one furlong is equivalent to approximately 201 metres). In jumps racing, the maximum amount of use was eight times during the race, five times after the last obstacle [15]. In terms of penalties, jockeys were to forfeit their prize money and riding fees if they received a suspension of three or more days. A range of suspensions were imposed for jockeys depending on how many times they exceeded the limit. The jockeys argued the rules were unmanageable and the penalties too severe. As a result, they threatened to strike. This action was averted by amended rules implemented on 21 October, which removed the restriction on the number of times the whip could be used in the last furlong (flat) and after the last obstacle (jumps), but the maximum limits of seven (flat) and eight (jumps) still applied [16]. Additional rule amendments occurred on 10 November and then finally on 21 February, 2012. In these amendments, stewards decided if fines and suspensions should occur, rather than it being an automatic breach. And, if jockeys hit the horse one or two times over the limit these were viewed as minor breaches, rather than being a cause for incurring an automatic fine [17].

In Australia, the amended rules AR137A were implemented on 1 August, 2009. The rules considered the most controversial by owners, trainers and jockeys were Rule (5) and (6):

In a race or trial a rider using the whip must give his (sic) horse time to respond and, other than on one occasion in the last 200 m when the whip may be used in three consecutive strides, the whip shall not be used in consecutive strides, other than in a slapping motion down the shoulder, with the whip hand remaining on the reins, or alternatively in a backhand manner.

A rider shall not use the whip more than 5 times prior to the 200 metres other than with a slapping motion down the shoulder, with the whip hand remaining on the reins, or alternatively in a backhand manner.

[18]

Following strike action by jockeys in September 2009, rule AR137A 5b was introduced on 26 September so that “in the final 100 metres of a race, official trial or jump-out, a rider may, subject to the other requirements of this rule, use his (sic) whip at his discretion” [19]. This means that there are no restrictions on the number of times a whip can be used in the last 100 metres in Australia. The limits in 2009 applied to the use of the whip in a forehand manner, not backhand application. New rules were introduced on 1 December, 2015, with Racing Australia deciding “to remove the distinction between forehand and backhand whip strikes so that there is a limit of five forehand or backhand strikes prior to the 100 metres” [20].

## 3. Human-Horse Relations and Media Framing

Media framing provides a framework to explore multiple discourses and different perceptions relating to a particular issue. There are varying definitions of and applications of framing analysis [21]. Entman argues that the definition of framing is “selecting a few aspects of a perceived reality and connecting them together in a narrative that promotes a particular interpretation” ([22], p. 391). For Cacciatore *et al.* “media frames act as organizing storylines for news issues, suggesting to audiences what is relevant about an issue and what can be ignored”, with articles often weighted towards one side of the debate ([23], p. 1041). Frames are, therefore, “at the core of an interpretative package” ([24], p. 438). Combined with Critical Discourse Analysis (CDA), this form of analysis provides a means of “linking media text and context” ([25], p. 406), a useful tool in exploring how human–horse relations are constructed in both print and social media.

The analysis of media articles has been utilized effectively to explore the different perceptions relating to jumps racing (a controversial sport due to a higher rate of injuries and fatalities to horses compared to flat racing). McManus and Montoya investigated how the relationship between human and non-human actors in jumps racing is represented and negotiated in the media and how this shapes the sport and influences equine welfare [25]. Media, therefore, is “an important part of the dynamic assemblages that support particular perceptions and treatment of animals” ([25], p. 403). This account reveals how proximity to a horse plays an important role in who gets to “speak” for the horse, with the opinions of those outside of the industry regarding equine welfare often dismissed as being irrelevant. McManus, Graham and Ruse, expand this discussion by highlighting the diverse messages conveyed in print and social media following the deaths of Yangming and Roberty Bob in a New Zealand jumps racing meeting in 2013 [26]. While the print media “tended to downplay” these events, activists utilized social media to draw attention to what they considered a major welfare issue ([26], p. 17) Social media, therefore, provided a space for people with alternative views to those involved in the racing industry to present their position, thereby disrupting “the narratives of traditional print media” ([26], p. 17).

The way that people consume, reflect and engage with current affairs is changing with people now reading more news online than in print [27]. Articles posted on newspaper websites tend to follow the same structure as print media. What makes these sites different is that they, like a multitude of online media sites, provide platforms for people to connect on a global scale and debate issues [23]. Social media sites reveal different styles of communication and language than conventional print media as they are often not restricted by the same editorial controls. Some newspaper websites, however, do not allow for online comments with others requiring users to be subscribers before posting comments thereby enforcing some control over online content. These sites often offer anonymity, with participants using pseudonyms, which can provide a more frank and open debate over particular issues [28]. Cacciatore *et al.* argues that the internet provides an interactive space in which a heterogeneity of opinions are expressed; however, “very few blogs actually reach members of the public, and most are partisan in nature”, which “may not represent the diversity of opinions surrounding an issue” ([23], p. 1043). The same could be said of online readers’ discussions, where the spaces provided only allow for brief comments, which often result in “incomplete snippets of argument, oblique and sometimes confusing opinions that vent but do not inform” ([29], p. 23). Limitations therefore apply to both print and social media, yet both are powerful instructive sites to view the creation and distribution of a variety of perspectives relating to animal welfare.

## 4. Research Methods

A preliminary search was undertaken using Factiva (media archival search engine) to locate articles in the print media relating to the whip debate relevant to the UK and Australia. The UK (2011–2012) search identified that the majority of the articles were in April, June, September–November 2011 and February 2012. These dates represented discussions about the introduction of new rules, amendments and major whip infringements by two jockeys. A similar search was undertaken for the period August 2014–August 2015. Newspaper articles were downloaded into a word format with each relevant print media story recorded in an excel spreadsheet by newspaper, date, author, headline, key words and phrases, and details of who was quoted. A Google online search was also undertaken with the same dates and search terms as the print media. The search revealed comments to online newspaper articles and posts on other social media sites. This exercise was repeated for the Australian print and social media, with the key periods being March–September 2009 and August 2014–2015.

Due to the large volume of articles in the UK newspapers (April 2011–February 2012), attention was focused on newspapers with more than five stories (see Table 1). This realized nine newspapers, with 189 stories. The majority of the information (88 percent) was in the racing and sports sections, with 86 percent being articles. For the period 2014–2015, the search located 35 stories, from eight newspapers (refer Table 1 below). The largest contribution was from *The Racing Post* (this publication was not available via Factiva for the 2011–2012 search). The majority of the 2014–2015 stories were located in the sports section (69 percent), with increased number of articles in the news (17 percent) and letters sections (14 percent), principally in *The Racing Post*.

Table 2 provides a breakdown of the Australian print media (March–September 2009) and (August 2014–2015). In 2009, there were 175 stories in nine newspapers, with the majority (62 percent) being articles in the sports section. Letters accounted for twenty-five percent, news six percent and the remaining seven percent were in the features and opinions. The largest contribution of letters (19 from 43) was published in the *Herald Sun.* The 2014–2015 search revealed 29 stories, the majority (97 percent) were articles in the sports section.

For the purposes of this research, social media was any article posted online which had online readers’ comments. This included newspaper and other websites that posted content about the whip debate. The search revealed 15 UK sites (2011), with 510 online readers’ comments, of which 272 were relevant to the whip debate. Only one site was located in the 2015 search, with ten relevant comments. In Australia (2009) 88 posts, of which 62 were relevant, were sourced from four sites. The 2015 search revealed similar levels with five sites located and 54 relevant comments from 93 posts. Multiple posts by authors were included unless they were a repetition of earlier posts. The relevant online readers’ comments were numbered sequentially and coded on the same basis as the print media.

## 5. Framing of Human-Horse Relationships in Media Coverage of Whip Debates

The media frames identified in this study were informed by previous studies on media analysis of issues relating to equine welfare [25,26,30]. All articles were read several times which identified three key themes and eleven frames (some specific to the UK, others unique to Australia, and frame eleven appearing only in online readers’ comments). The welfare theme related to whether the use of the whip was perceived as an issue or a problem, and who should make decisions about its use (relevant frames 1, 2, 3, 7, 9, 11); the discussion regarding the rules, regulations and penalty structures was categorized as a governance theme (frames 5, 6, 8, 10); and the economic theme related to discussions about gambling (frame 4). The frames are presented below:

**The Whip as a Tool (1).** This frame is predominately adopted by key actors involved in racing administration, racing insiders, and supported by racing journalists, enthusiasts and punters. In this frame, the whip is an essential tool in horse racing, exemplified by John Schreck (former Australian Racing Steward); “Let us not forget that a whip properly used by race riders is a tool that enhances the performance of racehorses”[31]. From this perspective, the whip is not cruel as it does not cause pain: “it is like hitting something with a rolled-up newspaper” [32]. The major problem with the whip is not how and why it is used, but how it is perceived by the “public”. As Tom Scudamore (UK jockey), writes:

Years have been spent on the development of the whips that we now use, with findings that promote the use of the stick. Yet instead of backing these findings, we are pandering to the ignorance of perception rather than educating the ill-informed as to why the whip is a necessary aid.

[33]

**The Whip Should Be Banned, or Is Not Needed (2).** The opinions of two groups emerged in this frame. In one group, actors argue that the whip is cruel; it inflicts pain and should therefore be banned. In the words of John McCririck (UK Racing Pundit), “I find the whip repulsive. No animal should be allowed to be hit. You cannot whack your dog with a stick so why should horses be any different” [34]. This view is reflected in the Australian media by Patrick Smith, writing in *The Australian*: “Whipping is cruel. It is, by any definition, barbaric. Horses are forced to race and they are beaten by a stick to perform” [35]. The second group was of the opinion that the whip did not inflict pain, but it should be carried for safety purposes rather than being used on the horses. These two points were often mentioned in conjunction in the articles reviewed. This opinion appeared often in relation to Towcester racecourse’s (UK) proposal to hold whip-free racing (refer Frame Ten).

**Who Knows Best? (3).** The welfare of thoroughbred horses is extremely important to a diverse group of actors. In this frame, people who work with the horses on a daily basis (trainers, jockeys, owners) state that they know what is best for the care of their horses. From this perspective, people who are not involved in the industry should have no influence over how the business is operated. This is highlighted in an article by Matt Stewart, racing journalist, who refers to the industry as a “racing bubble” arguing that:

There is a damaging mood in racing that the world outside the bubble is wrong and that racing is right; that racing can prosper quite nicely, thank you, without being influenced by agendas of politically correct hippies who wouldn’t swat a mosquito.

[36]

Those opposed to the whip were often referred to as being “anti-racing” and in the minority as detailed by A P McCoy (UK Jockey):

What worries me is that the views of a small minority of people, many of whom are anti-racing, anti-everything, are driving this and their views are given equal weight to those who work with horses 24 h a day, seven days a week. Eight million watched the Grand National, 100 or so wrote in to complain about the whip. Should that make it racing’s biggest problem? Not in my book.

[32]

This frame did not appear to the same extent in 2015, possibly because there was not the same level of concern about the whip being banned.

**Gambling (4).** Gambling revenue is extremely important for the economic viability of horse racing [37]. And yet, this was a relatively minor frame. The main arguments were that punters would not bet on the races, or would limit their betting, if a rule was introduced that restricted the number of times the jockeys could use the whip in the last 100 metres (Australia) or last furlong (UK), if the horse was disqualified, or if the whip was banned. The view expressed was that punters’ expected to see the jockeys doing their best to win. Robbie Waterhouse (a former Australian bookmaker and husband of leading Australian trainer Gai Waterhouse), stated “everyone is talking about not wanting to bet unless they see horses ridden out with the whip. I think it is starting to have an effect on betting turnover” [38].

**Rules and Regulations (5).** This frame dominated the discussion in the UK (2011) and Australian media (2009). In the UK, this discussion focused on the dissatisfaction with BHA’s handling of the situation, especially the timing of the introduction of the rules (brought in five days before Champions Day—one of the UK’s iconic race meetings); the penalties imposed, the restrictions on whip use, whether the changes were needed, and the threat of strikes. An example is Lee:

Sympathy from the racing public has largely rested with the jockeys since the bungled introduction of the new rules last month. Very quickly, the penalties were seen as draconian and the restrictions as inhibiting the will to win. Memories being conveniently short, many even questioned why such reforms were needed.

[39]

Similar dissatisfaction with the rules also featured in the 2009 Australian media with Gai Waterhouse describing the new whip laws as:

The silliest rule(s) ever made … It is like telling John McEnroe he can hit the ball every second time, or telling a chess player you can’t use a pawn … Every punter, owner, breeder, trainer must be against the new rule.

[40]

**Punishment Doesn’t Fit the Crime (6).** (UK specific 2011): This frame is exemplified in a quote by Paul Webber (trainer), “The penalties can’t have been thought through carefully enough because the punishments don’t fit the crime” [41]. This argument was time-specific, appearing in the UK media up to the beginning of December 2011, around the time when the penalties were reduced to satisfy jockeys and trainers. This frame also features a second argument, which stated that horses should be disqualified if the jockey breached the whip rule (this is the major topic of discussion in 2015). As highlighted by Barnes, “at the heart of the problem is the fact that jockeys are punished for hitting horses, but not owners and trainers” [42]. This frame did not appear in the Australian media in 2009 as the major point of contention was over how many times the whip could be used. The Australian amended rules did not set fixed penalties. In the UK, the rules changes were accompanied by a Guide for the stewards that detailed the penalties for offences. This frame does, however, emerge in the Australian media in 2015 as an integrity issue (see Frame Ten). It does not feature to any large extent in the online readers’ comments.

**The Horse (7).** This was a relatively minor frame, where individual horses were spoken of and where human-like qualities were ascribed to the horses. In this context, some horses were referred to as being “lazy” and therefore needed to be whipped, as reflected in a quote by Bart Cummings (legendary Australian trainer), “The whip is essential for non-performers to achieve success … The good ones don’t need it; the ordinary ones do. Unfortunately, there are more ordinary ones than good ones” [43]. This was in contrast to human qualities given to the so-called “good” horses. For example, Frankel, an “icon” of UK flat racing, was positioned as “an honest horse”, with a “lot of courage” [44].

**Whip-Free Racing (8).** (UK specific 2011): This minor frame offers a possible solution to the use of the whip. The committee from the Towcester Racecourse (south-west England) proposed trialling whip-free racing. This was heralded by some as a great idea, but dismissed by others. In this frame, Norway was mentioned as an example of a country that had successfully limited whip use. This was, however, dismissed by some as being irrelevant to the British racing scene. This frame was not evident in 2015.

**Community Values and Change (9).** Patrick Smith, writing in *The Australian*, produced the most articles in this frame arguing that the racing industry needs to change. For example, “our community values have changed and racing must change with them to stay tolerated, never mind relevant” [45]. This was a relatively minor frame in Australian media, and was even less evident in UK media.

**Integrity (10)** (Australia-specific). This frame is similar to that expressed in the UK under frame six, in that a horse should be disqualified/relegated if a jockey breaches the rules, but it was framed as an integrity issue in Australia. John Messara (owner-breeder,) argues that the “integrity of racing could be compromised” if rules were introduced that disqualified the horse: “how will the owner of a horse beaten by another horse whose jockey breaks the rules be compensated?”[46]. This was an issue in the print media in 2009, but only a minor discussion in 2015. It did not appear in social media.

**Ban Horse Racing (11).** This frame appeared only in the online readers’ comments of a website administered by Care2.com Incorporated (the world’s largest community website) (2011) in a discussion about whip use in horse racing.

### Frequency of Media Frames in Whip Debates

Following from the above identification and elaboration of media frames, it is important to quantify the occurrence and frequency and to situate the use of media frames in both the UK and Australia. Table 3 decomposes the total UK newspaper coverage into the nine relevant frames from the eleven frames discussed above. The focus of attention in the print media (UK 2011–2012) was on the “rules and regulations” (30 percent of the frequency of total coded frames). This reduced in volume once the BHA amended the rules, principally to satisfy the jockeys and trainers, and was no longer an issue in 2014–2015. In the Australian print media (refer Table 4) this frame remained at similar levels over the two time periods (2009 39 percent; 2014–2015 33 percent). In the UK example, there was an increase in frequency relating to ‘punishment doesn’t fit the crime’ (2011–2012 26 percent; 2014–2015 57 percent) as the discussion changed from the unfairness of the penalties to whether the horse should be disqualified if jockeys breached the rules. The other significant frame, in terms of frequency, was “who knows best” and this retained similar levels between the two periods relating to the UK. In Australia, there was a slight increase from five percent in 2009 to 12 percent in 2015, as a result of the discussion about the 2015 whip rules.

With regards to the debate about the use of the whip, in the print media (UK 2011–2012) there was a higher frequency of the “whip as a tool” frame (18 percent), compared with “whip should be banned” (five percent). This is not surprising as the majority of the articles in the print media were in the sports section (88 percent) and the majority of the people quoted were from the racing industry (89 percent). An additional seven percent were quotes from the RSPCA (UK), whose position is that there is a place for the whip in racing as long as it “used primarily for safety, correction and encouragement purposes” [47]. This is a far more moderate approach than Animal Aid, who want the whip banned, and who were not quoted in the 189 articles reviewed. This organization did, however, have an opinion piece printed in *The Guardian* (November 2011). It is therefore, not surprising that there is a difference, in terms of frequency, in relation to these frames. In 2014–2015, there is more of a balance between these frames with one possible reason being that more letters appeared in this print media search.

Similar to the UK, the majority of people quoted in Australian print media (2009) were people involved in the racing industry (88 percent). There was, however a difference in relation to “the whip should be banned”, which had a higher frequency of 18 percent compared to 11 percent, “the whip as a tool”. One reason for this is that there were more letters in the Australian print media (twenty-five percent of the total number of documents analyzed) than the UK (2011) print media (2 percent), with the majority of the writers calling for the banning of the whip. Similar to the UK, the Australian RSPCA was quoted. Based on the quotations attributed to the RSPCA, the organization had a more moderate approach than their current stance of wanting the whip banned. There were no quotes attributable to animal rights groups.

In social media the call to ban the whip is the dominant frame in terms of frequency (39 percent of the total number of analyzed frames—refer Table 5), especially in the UK (2011–2012). This is mainly due to the large number of contributors on the Care2.com website, which asked the question “how many time should a jockey whip a racehorse?” [48]. It was on this site that the frame calling for horse racing to be banned also emerged. The volume of discussion reduces in 2014–2015, as there are no longer any key issues being debated in the media. This is also the dominant frame in the Australian context (refer Table 6), but the issue is still being debated over the period (August 2014–2015). The principal reason for this is due to the airing of “Horse Whip” (24 March 2015), part of the ABC Television’s Catalyst program, in which it was suggested that the whip was “likely to be painful” to horses [49].

## 6. Media Perceptions and Equine Welfare

This paper advances the work of McManus, Graham and Ruse in which they argued, in the New Zealand context, that “some social media sites led to the creation of alternative narratives than in print media” ([26], p. 197). This observation is borne out and extended in the present study in the Australian and UK contexts. The results outlined above indicate a difference in print and social media framing of the debate about the use of the whip in horse racing. Journalists in the print media were more likely to obtain comments about the whip debate from people who supported the use of the whip. In contrast, those opposed to the whip found their voices in social media sites. The perspective of those wanting the whip banned was less visible in print media, with the exception of letters to the editors. Online media sites thus appear to provide an alternative space for people to express their opinions. Journalists are “gatekeepers” as they are able to select what information will or will not be published in print and associated online media content [50]. Often, it is not what they explicitly write, but who they quote, that influences the tone and argument of the article. Commentators posting in online media sites are not restricted by the same editorial controls as journalists, with many newspaper organizations facing the challenge of how to moderate online comments to their articles [51].

This paper has confirmed that the principal perceptions of the advocates of the whip, as portrayed in conventional and social media in Australia and the UK, were that the whip was an essential aid that did not harm horses. Those opposed to the whip argued that it inflicted pain. The whip debate did not change to any large degree between the two countries and over the time periods investigated. What did change was the volume of discussion, which reduced in most cases, except in the case of the Australian social media where it remained at similar levels, principally due to the airing of “The Whip” [49]. Media issues often have “an attention cycle” then over a period of time the debate diminishes ([23], p. 1041). In the UK (2011) and Australian (2009) whip debate, the cycle was constantly being extended as rules were amended, resulting in intense media activity. In December 2015, Racing Australia implemented new rules. It was initially suggested that jockeys were considering fighting the proposed changes [52]. This did not occur with print media attention moving onto the next big racing story.

Proximity to the horses influences who “speaks” for the horses and who is therefore considered by the print media to be the “legitimate voice” in equine welfare matters ([25], p. 145). People inside the racing industry—the owners, trainers and jockeys—who were quoted in print media were positioned as “experts” and therefore knew what was best for the care of the horses. The horse was spoken of by those working with them closely as both a “loved” and working animal, highlighting that it is possible for emotionality and instrumentality to co-exist in equine industries [30]. This is reflected in quotes by two jockeys who ride in the UK. The first by Richard Hughes, “I love horses and would never abuse them” [53]. And, Ruby Walsh “horses are animals and animals can’t be treated as humans. Race horses are a specific breed to do a specific job. They’re not pets” [54]. The emphasis in print and social media sites, which were positive about horse racing, was on the relationship between the jockey and the horse as a partnership. Very little recognition was given to ideas that equine-human relationships are often about power, with humans controlling what happens with horses during their racing career [55,56]. The power relationship was more implicit in online media, where the relationship was portrayed as exploitative. For example, horses were spoken of as “slaves, pure and simple. and their purpose is to entertain. and then die” (Sic) [48]. And conditional, “I understand that punters, owners and trainers love horses, but that does not extend to when they become a financial burden, then there is another younger, fitter horse to love” [48]. 

Atkinson and Young highlighted the presence of a “historically pervasive view that animals neither experience pain as humans do (*i.e.*, reflexively or emotionally) nor should they be considered sentient entities with moral rights” ([3], p. 343). This type of argument was present in some of the online discussions. For example, in comments posted in response to Dean Stansall’s (Animal Aid) comment piece in The Guardian, “there is absolutely no evidence that a horse feels pain, it is only the whoosh sound that makes horses go faster … the new air-cushioned whips never inflict weals” [57] (a point disputed by opponents of the whip). An alternative perspective was also displayed in social media, with one online user writing “animals are not made out of plastic or tin, just like humans they feel pain and if its (sic) cruel to whip a horse off the track then its (sic) cruel to whip them in a race” [58]. There was, however, also some who condoned the excessive whipping of the horses, seeming to suggest that it did not matter if it caused pain or not. Two online users, posting in 2012 on the YouGov UK website, wrote, “seeing jockeys lay into their horse whipping them furiously adds to the excitement of the race” [59] and, “most horses need to be thrashed to get the best performance out of them. Also punters like aggressive riders like me cos (sic) they want to see the horse getting a good whipping to get their money’s worth” [60]. This view was supported by another seven out of the ten contributors, but this was the only site where this discussion thread emerged. Alan Lee, writing in The Guardian refers to these types of comments as being produced by people with a “betting shop mentality”, where:

Wild-eyed punters clutch their betting slips like starving men with meal tickets and unconsciously ride a finish with their gaze fixed on the screens. Falls of rival horses are cheered and jockeys are exhorted to “hit the thing” until defeat has them routinely denounced as crooked and useless … But the world outside the betting shop has moved on and racing can no longer be seen as compliant in such crassness.

[61]

Changing social values and emergent technologies have influenced animal welfare, with recognition that sports where animals are killed are no longer acceptable [62]. For the whip to be banned there needs to be another cultural shift where any sport that has the potential to harm animals is considered unacceptable.

## 7. Conclusions

The thoroughbred racing industry is a socio-cultural assemblage of diverse actors with differing world views [5]. This research, however, reveals that the majority of the people involved in the industry who were quoted, or wrote articles, in print media held similar perceptions about the use of the whip and who should play a role in the care of the horses. Narratives about the horses and their care were created and reinforced in this media context. The majority of the stories were authored by racing journalists, in the racing and sports section of the paper, who were mostly supportive of the racing industry. Interviews with people from inside the racing industry dominated.

This research highlights that the opinions of those outside the “racing bubble” are being expressed in various social media forums, with the conversations in online forums focused on whether the whip should be banned or not. The racing industry has implemented changes that have improved equine welfare, including introducing padded whips and new whip rules (which were amended to satisfy those who resisted the changes). While the industry is aware that some people believe that the whip does hurt, this is often deflected with the argument that the public do not fully understand how and why the whip is used.

Jones *et al.* have called for independent research to prove the pain-free status of the whip and its “usefulness in safety” [13]. This will not satisfy all actors in the debate, as there are groups that will never support an industry they consider to be exploiting animals. However, it may be one step towards acceptance by the public if they believe the industry is doing everything possible to ensure the welfare of the horses. People outside of the “racing bubble” will keep having their say on what they consider to be acceptable equine practices, increasingly through social media. This media platform, therefore, plays a significant role in the treatment of animals by conveying different perceptions about equine welfare and the positioning of the horse.

## Figures and Tables

**Table 1:** UK print media: 2011–2012 (and 2014–2015 in brackets).

The Guardian	48 (4)	Daily Mail	9
The Times	34 (2)	The Journal-Newcastle	9
The Telegraph	29	The Racing Post	(22)
The Independent	19	The Weekender	(3)
Daily Mirror	18 (1)	Trinity Mirror	(1)
The Sun	16 (1)	Western Morning News	(1)
The Belfast Telegraph	7		
**Total 189 (35)**			

**Table 2:** Australian print media 2009 and 2014–2015 (in brackets).

Sydney Morning Herald	34 (7)	The Age	15 (2)
Daily Telegraph	31	Advertiser	15
Herald Sun	29 (2)	Sportsman	5 (2)
The Australian	26 (7)	West Australian	4 (1)
Courier Mail	16	Miscellaneous (8 newspapers, with 1 article each)	(8)
**Total 175 (29)**			

**Table 3:** Frames in UK Print Media: April 2011–February 2012 and (August 2014–August 2015).

Frames	One	Two	Three	Four	Five	Six	Seven	Eight	Nine	Total
	The Whip as a Tool	The Whip Should Be Banned	Who Knows Best	Gambling	Rules & Regulations	Punishment Doesn’t Fit the Crime	The Horse	Whip-Free Racing	Community Value and Change	
Times	10	2	11	1	9	20 (2)		2	1	
Guardian	9	2 (1)	7 (1)	1	22 (1)	17 (1)		2	(1)	
Sun	3 (1)	2	3 (1)		5	10	3 (1)	1		
Telegraph	5	1	2	1	22	14	1	3		
Daily Mirror	8		1	3	9	3	3			
Independent	6	1	1		8	6	3	1		
Journal-Newcastle	2	1	1	1	5	3	2			
Daily Mail	6	6		1	5			2		
Belfast Telegraph	5	1	1	1	3	2	1			
Racing Post	(1)	(2)	(3)	(2)		(17)	(1)		(2)	
Weekender	(1)	(1)				(5)				
W M News						(1)				
Total	54 (3) 18% (7%)	16 (4) 5% (9%)	27 (5) 9% (11%)	9 (2) 3% (4%)	88 (1) 30% (2%)	75 (26) 26% (56%)	13 (2) 4% (4%)	11 4%	1(3) 1% (7%)	**294 (46)**

**Table 4:** Frames in Australia Print Media: 2009 and (August 2014–2015).

Frames	One	Two	Three	Four	Five	Seven	Nine	Ten	Total
	The Whip as a Tool	The Whip Should be Banned	Who Knows Best	Gambling	Rules & Regulations	The Horse	Community Values & Change	Integrity	
Sydney Morning Herald	4 (3)	8 (1)	4 (1)	8	28 (4)	5 (1)	3(2)	3 (1)	
Daily Telegraph	1	7	1	2	15		4	6	
Herald Sun	12 (1)	11 (1)	2 (1)	1	9	2	1(1)	3	
Australian	2 (1)	9 (4)	6 (3)	2	22 (6)	5	12 (1)	1	
Age	1	3 (1)			9 (1)		1 (1)	(1)	
Sportsman	(1)		(1)		5 (2)	2	1	1	
Advertiser	3	4		1	9	2	2	2	
Courier Mail	2	3		2	8	1	1		
West Australian	4 (1)	2	1		2 (1)				
Miscellaneous	(2)	(3)	(1)		(5)	(2)	(1)	(1)	
Total	29 (9) 11% (16%)	47 (10) 18% (18%)	14 (7) 5% (12%)	16 6%	107 (19) 39% (33%)	17 (3) 6% (5%)	25 (6) 9% (11%)	16 (3) 6% (5%)	**271 (57)**

**Table 5:** UK: Online readers’ comments April 2011–February 2012 (2015 not noted as only ten comments from one site).

Frames			One	Two	Three	Four	Five	Six	Seven	Eight	Eleven	Total
Date	Source	No of Relevant Comments (Total)	The Whip as a Tool	The Whip Should Be Banned	Who Knows Best	Gambling	Rules and Regulations	Punishment Doesn’t Fit the Crime	The Horse	Whip-Free Racing	Ban Horse Racing	
21/4/2011	Telegraph	4 (10)		2		2			2	2		
23/4/2011	Daily Mail	10 (10)	2	6	1	1		2		3		
26/4/2011	Guardian	4 (8)		1			2	1	1			
27/9/2011	Daily Mail	7 (7)	4	1	1	1						
30/9/2011	Guardian	5 (6)		4		1	1			1		
September 2011	H & H	16(36)	9	7	1		5	1				
October 2011	H & H	8 (15)		3	4							
28/10/2011	Betfair	2 (2)	2				1					
8/11/2011	Guardian	20 (25)	6	5	5		1		4			
11/11/2011	Betfair	20 (86)		2	18	1	1					
21/11/2011	YouGov.UK	10 (10)	7	2	1	2		1	2			
27/11/2011	Care2care	125 (179)	6	93	2	2	1		12		37	
21/2/2012	Betfair	31 (96)	6	3	20	3			1			
21/2/2012	Telegraph	1(2)	1					1				
23/2/2012	Guardian	9 (18)	3	3	3	1	1	1	3			
		272 (510)	46 (14%)	132 (39%)	56 (17%)	14 (4%)	13 (4%)	7 (2%)	25 (7%)	6 (2%)	37 (11%)	**336**

**Table 6:** Australia online readers’ comments 2009 and August 2014–2015.

Frames			One	Two	Three	Four	Five	Seven	Total
Date	Source	No of Relevant Comments (Total)	The Whip as a Tool	The Whip Should be Banned	Who Knows Best	Gambling	Rules & Regulations	The Horse	
30/8/2009	Sunday Telegraph	57 (64)	33	24	8	4	1	1	
11/9/2009	Animals Australia	2 (20)		2					
24/9/2009	RSPCA	1 (1)		1					
1/8/2009	Indymedia	1 (3)		1					
	**Total 2009**	61 (88)	33 (45%)	28 (37%)	8 (11%)	4 (5%)	1 (1%)	1 (1%)	75
24/3/2015	ABC	18 (52)	4	14		1	2		
30/10/2015	Sydney Morning Herald	12 (14)	5	7					
25/2/2015	The Australian	3 (5)	1	2					
28/7/2015	Thoroughbred Village	4(5)		2			2		
27/4/2015	Racenet	17 (17)	4	7	2	2	2	2	
	**Total 2015**	54 (93)	14 (25%)	32 (54%)	2 (3%)	3 (5%)	6 (10%)	2 (3%)	59
